# Computational Drug Repurposing Across the Multiple Myeloma Spectrum: From MGUS to MM

**DOI:** 10.3390/cancers17183045

**Published:** 2025-09-18

**Authors:** Kyriaki Savva, Marilena M. Bourdakou, Dimitris Stellas, Jerome Zoidakis, George M. Spyrou

**Affiliations:** 1 Bioinformatics Department, The Cyprus Institute of Neurology and Genetics, Nicosia 2371, Cyprus; 2 Institute of Chemical Biology, National Hellenic Research Foundation (NHRF), 11635 Athens, Greece; 3 Biotechnology Division, Biomedical Research Foundation, Academy of Athens, 10447 Athens, Greece; izoidakis@bioacademy.gr; 4 Biology Department, National and Kapodistrian University of Athens, 10679 Athens, Greece

**Keywords:** drug repurposing, multiple myeloma, prodromal stages, drug combinations

## Abstract

Background/Objectives: Multiple myeloma (MM) is a challenging, B cell malignancy characterised by the uncontrolled proliferation of plasma cells within the bone marrow. Despite significant advances in treatment options nowadays, MM remains an incurable malignancy, with the majority of patients succumbing to the disease. MM develops from a pre-malignant state known as monoclonal gammopathy of unknown significance (MGUS), which then has the potential to evolve either into smouldering (asymptomatic) multiple myeloma (SMM) or into MM. Since novel drug discovery takes years to reach the clinic, drug repurposing, which concerns the detection of existing drugs for a novel disease, can be applied. Methods: To address this critical and still unmet medical need, we present a comprehensive signature-based drug-repurposing approach using all the publicly available bulk transcriptomics datasets on mGUS, sMM, and MM. Results: Our study included an in-house scoring scheme approach enabling further filtering and prioritisation, resulting in 25 candidate repurposed drugs for mGUS, 23 for sMM, and 66 for MM. The corresponding gene targets and the related functional terms have been analysed, providing extra information for stage-specific underlying mechanisms in myeloma. Lastly, enabled by a specific computational workflow, we propose drug combinations between our top candidate repurposed drugs and FDA-approved drugs for MM. Conclusions: Together, these results deliver a stage-specific, transparent resource for MM drug repurposing and combination design, intended to accelerate translation toward earlier disease intervention and improved patient outcomes.

## Simple Summary

Multiple myeloma is a cancer of plasma cells in the bone marrow. It often develops gradually, starting from a harmless stage called monoclonal gammopathy of undetermined significance, then a higher-risk stage called smoldering multiple myeloma, and finally symptomatic disease. New medicines usually take many years to develop, so finding new uses for existing medicines can bring treatments to patients faster. Our goal was to discover medicines that could help at each stage of this disease. We analysed public studies across these three stages and used an approach to match disease patterns with medicines that might reverse them. We identified 25 candidates for monoclonal gammopathy of undetermined significance, 23 for smoldering multiple myeloma, and 66 for multiple myeloma. We also examined the biological pathways these medicines affect and suggested combinations with current treatments. These results provide a stage-specific resource to guide laboratory testing and clinical exploration, with the aim of improving earlier intervention and outcomes for people at risk of, or living with, multiple myeloma.

## 1. Introduction

Multiple myeloma, the second-most common haematological malignancy after non-Hodgkin lymphoma, originates from terminally differentiated plasma cells primarily found in the bone marrow. The disease is characterised by the secretion of a monoclonal immunoglobulin protein (M protein) in most patients, driving clinical manifestations such as hypercalcemia, renal insufficiency, anaemia, and bone disease [1]. Monoclonal gammopathy of undetermined significance (MGUS) represents an asymptomatic precursor to multiple myeloma, involving clonal plasma cells and M protein secretion. Approximately 15% of MGUS cases progress to multiple myeloma over 25 years, and early detection has shown positive effects on overall survival [2]. Smouldering multiple myeloma (sMM) serves as an intermediate stage between MGUS and MM, with recent updates considering ultra-high-risk sMM as part of multiple myeloma and suggesting that early treatment for high-risk sMM may delay progression to full-blown MM [3]. More specifically, MGUS is an asymptomatic clonal plasma cell disorder (M protein < 3 g/dL; marrow plasma cells < 10%; no common symptoms of MM). Progression to MM averages ~1%/year but varies with isotype, M protein level, and free light chain ratio [4]. sMM shows higher tumour burden (≥10% marrow plasma cells and/or higher M protein) without end organ damage; risk stratification identifies subsets for early intervention [5]. Symptomatic MM is defined by clonal plasma cells (≥10%) plus myeloma-defining events, reflecting genomic lesions and microenvironmental cues driving proliferation, immune evasion, and bone disease [6]. Established diagnostic and prognostic biomarkers in MM include detecting M protein, free immunoglobulin light chain, β2-microglobulin and albumin, creatinine and calcium, and FISH analysis for cytogenetic abnormalities. These biomarkers are used in the disease’s staging system. Additionally, imaging is also used to detect the degree of bone marrow infiltration. Emerging diagnostic biomarkers include next-generation sequencing and next-generation flow cytometry, extracellular vesicles, miRNAs, and circulating tumour cells [7].

Current management includes regular monitoring for MGUS and low-risk sMM, which avoids drug toxicity but carries a small ongoing risk of progression [4]. In selected high-risk sMM, early treatment (e.g., daratumumab- or lenalidomide-based) can delay progression and improve outcomes, but increases infection risk and cost, and patient-selection criteria are still evolving [8]. In symptomatic MM, proteasome inhibitors (bortezomib, carfilzomib, ixazomib) yield high response rates, but neuropathy with bortezomib and cardiopulmonary events with carfilzomib remain key limitations, alongside eventual resistance [9]. Immunomodulatory drugs (IMiDs; lenalidomide, pomalidomide) provide durable benefit and underpin maintenance therapy yet cause cytopenia and venous thromboembolism [10]. Anti-CD38 monoclonal antibodies (daratumumab, isatuximab) achieve rapid cytoreduction and synergise with proteasome inhibitor and IMiD-based backbones, but are associated with infusion-related reactions, infections, hypogammaglobulinemia, and higher treatment cost [11]. BCMA-directed therapies—including CAR-T cells, bispecific antibodies, and antibody–drug conjugates—can induce deep remissions in relapsed/refractory MM, yet are limited by cytokine-release syndrome, immune-effector cell-associated neurotoxicity, infections with prolonged hypogammaglobulinemia, manufacturing/access constraints, and relapse driven by antigen escape [11].

Early standard treatment for MM included alkylating agents such as melphalan and/or cyclophosphamide, combined with corticosteroids in most cases. This treatment was followed by autologous stem cell transplantation, a standard treatment option still used today. Compounds such as thalidomide, lenalidomide, and pomalidomide also became available soon afterward. During the last two decades, the therapeutic pool of MM expanded even further, with the discovery of proteasome inhibitors (bortezomib), histone deacetylase inhibitors (panobinostat), and more recently, nuclear export inhibitors (selinexor). In addition, approval of immunotherapies for MM in 2015 starting with daratumumab and elotuzumab monoclonal antibodies and later on with the antibody–drug conjugate belantamab mafodotin, the bispecific teclistamab, and chimeric antibody receptor (CAR)-T cell products, such as idecabtagene and vicleucel, are standard treatment options nowadays. Despite the outstanding discoveries and improvements that were made regarding the treatment for MM, almost all patients become refractory to treatment and relapse. Because of that, there is a high need to detect or develop drugs for the treatment of relapsed and/or refractory MM or for halting the progression to MM [12]. One such approach is drug repurposing. Drug repurposing concerns the detection of novel indications for drugs that are already approved for another disease. Specifically, in silico drug repurposing offers the ability to detect potential drugs using computational methodologies in a cost and time-efficient way.

Motivated by the lack of a cure for MM or drugs that could halt the progression to MM, we focused on applying the avenue of computational drug-repurposing to highlight promising drug candidates and to study the underlying mechanisms of the drugs and pathways related to the disease and the stages that precede the disease. Since each MM stage (MGUS, sMM, and MM) is characterised by different symptoms and molecular mechanisms, we stratified the patient samples based on the different stages to pinpoint stage-specific candidate repurposed drugs.

Here, we present a novel approach to use the transcriptomic signatures of the molecular basis of the different stages of MM to detect candidate repurposed drugs per severity stage. Initially, we performed drug-repurposing analysis for different MM stages using publicly available transcriptomic data. Moreover, we show the pathways that our proposed repurposed drugs are involved in and their structural similarities with current clinical trial drugs of MM.

## 2. Objectives

- To analyse publicly available transcriptomic datasets across the full spectrum of multiple myeloma (MGUS, sMM, and MM) to identify stage-specific differentially expressed genes (DEGs) and perform pathway enrichment analysis.
- To apply a computational drug-repurposing pipeline aimed at identifying candidate therapeutic compounds for each disease stage and uncovering shared and distinct candidate drugs across the MM progression.
- To investigate the molecular targets and associated pathways of the repurposed drug candidates, offering insight into the biological complexity and heterogeneity of myeloma at each stage.
- To propose rational drug combination strategies, integrating FDA-approved treatments for MM with newly identified repurposed candidates.

## 3. Materials and Methods

**Figure 1:**
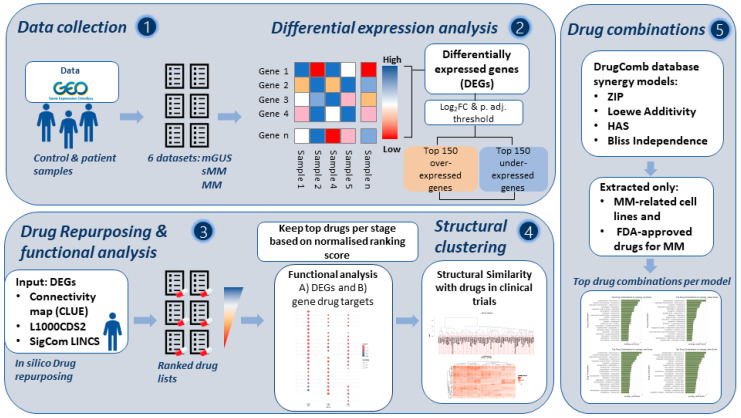
General pipeline of the study. Step 1: Transcriptomics data collection from Gene expression omnibus (GEO). Step 2: Pre-processing of data and detection of differentially expressed genes in R using the limma R package. Step 3: Transcriptomics-based drug repurposing—3 different tools were used: Connectivity Map (CMap) CLUE, L1000CDS^2^, and SigComLINCS. The top 150 over-expressed and 150 under-expressed genes based on log_2_FC from the gene list with an adjusted *p* value of <0.05 were used as an input. Gene ontology enrichment analysis of the differentially expressed genes and drug targets was followed. Kept the top pathways per dataset to keep a unique list for each stage using an in-house scoring scheme. Step 4: Collection of clinical trials drugs and structural similarity was performed for candidate repurposed drugs and drugs in clinical trials. Step 5: Proposal of drug combinations through 4 different models, using the FDA-approved drugs for MM and our proposed candidate repurposed drugs.

On 2 September 2024, we collected all the available bulk transcriptomic data of the 3 stages of MM progression: MGUS, SMM, and MM. We performed DE analysis per dataset and MM stage, using the Limma R package (version 3.64.0). Enrichment analysis of the DE genes and in silico drug repurposing using 3 different drug-repurposing tools then followed. After collecting the candidate repurposed drugs, we used an already established in-house scoring scheme to further filter the drugs. A structural comparison of the highlighted proposed drugs with the drugs that are currently in clinical trials was then followed. The targets of the proposed candidate repurposed drugs were found and enrichment analysis of those targets was then performed. Lastly, suggestions of candidate drug combinations of the MM FDA-approved drugs with our proposed candidate repurposed drugs were followed. The detailed pipeline is shown in Figure 1.

### 3.1. Data

Six different microarray MM datasets were retrieved from the Gene expression omnibus (GEO) [13]—a transcriptional data repository. Some of the datasets did not include all disease stages. The selection of the datasets was based on the disease staging: MGUS, sMM, and MM (Table 1). To our knowledge, these were the only publicly available datasets on the 2 September 2024 that clearly used this progression staging for the disease.

**Table 1:** The experimental design of the transcriptomic datasets used in this study. All stages were compared to controls.

No	Ref.	GEO Accession Number	Stage
1	[14]	GSE36474	MM
2	[15]	GSE5900	MGUS sMM
3	[16]	GSE6477	MGUS sMM MM
4	[17]	GSE13591	MGUS MM
5	[18]	GSE47552	MGUS sMM MM
6	[19]	GSE80608	MGUS MM

### 3.2. Pre-Processing of Data

Each dataset selected was quantile-normalised and log_2_-transformed where necessary. Subsequent analysis was performed in R statistical environment (http://www.R-project.org/, accessed 12 September 2024) [20]. Each of the six datasets and for each stage was processed using the Limma R package [21], a linear model that calculates a moderated t-statistic from gene expression experiments.

### 3.3. Detection of Differentially Expressed Genes

After the dataset pre-processing, probe-set IDs were matched to gene symbols according to each platform’s annotation file. We maintained the most differentially expressed ones in cases of gene symbol correspondence to multiple probe-sets (Tables S1–S6). Explicitly stage-labelled cohorts across the MGUS → sMM → MM spectrum are rare and span different microarray platforms with unequal stage composition. Thus, we adopted a conservative strategy: within-study normalisation and limma DE, followed by rank-level aggregation across studies per stage.

From the Limma analysis result, we kept the top 150 over-expressed and 150 under-expressed genes based on log_2_FC from the gene list with an adjusted *p* value of <0.05. All comparisons were made using the disease state (i.e., MGUS, sMM, or MM) vs. control samples. The selected number of genes (150 over-expressed and 150 under-expressed) corresponds to the input number limit of the drug-repurposing tools we used in the sequel. Many widely used signature-reversal tools perform best with balanced up/down lists of limited size. Using a fixed symmetric cutoff ensures comparability across tools and stages and focuses on the high-confidence perturbation while avoiding noise from long tails where directionality is least stable across datasets. To keep the study tractable and avoid tool-specific optimisation (which risks overfitting), we retained 150/150 as a pre-specified setting.

### 3.4. Pathway Analysis of DEGs

The Gene ontology (GO) enrichment analysis was conducted using the differentially expressed genes (DEGs) identified in the transcriptomics study. The focus was on biological processes (BPs), and the clusterProfiler R package (accessed on 18 April 2025) [22] was employed for the analysis. This procedure was applied individually to each stage and dataset, and a scoring method was used to combine the results, leading to a single list of pathways for each stage of the disease (Tables S7–S9).

To rank the pathways for each stage and dataset, leading to a unified list per stage, we ranked the pathways within each dataset according to their adjusted *p* values. Hence, the pathways from each dataset for each stage were combined as a union of unique pathways and ranked by calculating the weighted sum of normalised average rankings and the normalised number of appearances according to Equation (1):

(1) $${Score\;}_{i}=\;w_{1}\;*\;R_{i}\;+\;w_{2}\;*\;A_{i},\;i\;=\;1,\;\ldots ,\;N\;pathways\;$$

where R_i_ is the average ranking score from each of the three tools, A_i_ is the number of appearances of each pathway for the different datasets per stage, and w_1_ and w_2_ are set to 0.7 and 0.3, respectively. This scoring scheme was adapted from [23].

### 3.5. Transcriptomics-Based Drug Repurposing

The transcriptomic-based drug repurposing was performed using three different drug-repurposing tools: Connectivity Map (CMap) CLUE [24], L1000CDS^2^ [25], and SigComLINCS [26]. The 300 differentially expressed genes (based on their log_2_FC value) from the six different datasets were used as transcriptomic signatures. Next, each set was used as an input to the aforementioned repurposing tools. These tools use transcriptional expression data from multiple human cell lines to probe relationships between diseases and therapeutic agents. Drugs are sorted according to a score (inhibition score), which characterises if a drug can reverse (drugs with a strong negative score value) or mimic (drugs with a strong positive score value) the expression levels of a disease based on a given set of genes. For each stage and each dataset, we obtained a list of candidate repurposed drugs predicted by each of the three tools, ranked based on their inhibition score. Since the output of L1000CDS^2^ is limited to 50 drugs, we applied the same cutoff for all the other repurposed drug lists. Hence, the top 50 drugs from each of the three tools were combined as a union of unique drugs and ranked by calculating the weighted sum of normalised average rankings and the normalised number of appearances according to Equation (2):

(2) $${Score\;}_{i}\;=\;w_{1}\;*\;R_{i}\;+\;w_{2}\;*\;A_{i},\;i\;=\;1,\;\ldots \;,\;N\;drugs\;$$

where R_i_ is the average ranking score from each of the three tools, A_i_ is the number of appearances of each drug in the three DR tools, and w_1_ and w_2_ are set to 0.7 and 0.3, respectively. The drug lists obtained from all datasets were combined and re-ranked using Equation (1) to conclude a single drug list from all the datasets. This scoring scheme was adapted from [23].

After the scoring was completed, we retained drugs with a score of 0.75 or higher for each disease stage.

### 3.6. Collection of the Currently Running Clinical Trials of MM and Its Stages

All listed clinical studies related to the three stages were collected from www.clinicaltrials.gov, accessed on 5 January 2025. The downloaded file was filtered separately for MGUS, sMM, and MM and entries that did not match these terms were removed. Additionally, clinical trials that were either suspended, withdrawn, unknown, or terminated were removed in order to keep the entries that are active, completed, or will be recruiting soon. Specifically, only small-molecule drugs and biologicals were obtained from the studies, and everything else was removed.

### 3.7. Structural Similarity

The structures of the candidate repurposed drugs and the clinical trial drugs we collected were downloaded in the form of the Simplified Molecular Input Line Entry Systems (SMILES) through the PubChem Identifier Exchange Service of the PubChem database (https://pubchem.ncbi.nlm.nih.gov/idexchange/idexchange.cgi, accessed on 21 March 2025) [27]. We then converted the SMILES format into a single 2D structure data file (SDF) using the OpenBabel software [28]. The Rcpi R package (accessed on 27 March 2025) [29] was then used to perform structural similarity across the different drug groups collected, using an in-house script. We used an 80% Tanimoto similarity as a threshold. Additionally, we used a merged SDF of the shortlisted repurposed drugs of the three stages (MGUS, sMM, and MM) as input in the ChemBioServer 2.0 (https://chembioserver.vi-seem.eu/, accessed on 30 March 2025) [30], a publicly available tool that provides filtering, clustering, comparison of drug structures, and networking of chemical compounds to facilitate both drug discovery and repurposing. Drugs were clustered using the Soergel distance ≤ 0.15 corresponding to a Tanimoto similarity 87% (Tanimoto similarity = 1/(1 + Soergel distance)) [31].

### 3.8. Drug Target Pathway Analysis

To further explore the candidate repurposed drugs, we extracted the corresponding gene targets of each drug through mainly the Drug repurposing hub database (https://repo-hub.broadinstitute.org/repurposing, accessed on 30 March 2025) [32] per stage. In cases where no target was found, DrugBank (https://go.drugbank.com/, accessed on 30 March 2025) and PubChem (https://pubchem.ncbi.nlm.nih.gov/, accessed on 30 March 2025) were also used. (Tables S13–S15). The Gene ontology (GO) enrichment analysis was conducted using the drug target genes of the top candidate repurposed drugs, as chosen using the scoring scheme mentioned above. The focus was on biological processes (BPs), and the clusterProfiler R package was employed for the analysis. This procedure was applied individually to each stage, and the top pathways were kept using an adjusted *p* value of <0.05.

### 3.9. Drug Combination Synergies

The DrugComb database [33] was used to extract and analyse experimental drug combination data across multiple cancer cell lines. DrugComb (https://drugcomb.fimm.fi, accessed on 30 March 2025) is an open access data portal containing drug combination studies, which are standardised and harmonised. In total, 437,932 drug combinations were tested on a variety of cancer cell lines. The data were downloaded and filtered to include only MM-related cell lines and FDA-approved drugs for MM. The analysis was performed in R. Four synergy models were used:

- ZIP (Zero Interaction Potency): Measures interactions across different doses [34].
- Loewe Additivity: Compares observed combination effects to the expected additive effects.
- HSA (Highest Single Agent): Evaluates whether the combination is superior to the best-performing single agent.
- Bliss Independence: Assesses interactions based on independent probabilities of drug effects.

We used four complementary reference models. HSA benchmarks against the best single agent (conservative). Bliss assumes probabilistic independence. Loewe assumes dose equivalence (appropriate for similar mechanism pairs) and is typically most stringent when mechanisms diverge. ZIP integrates potency and effect shifts across the response surface. Therefore, model divergence, especially lower Loewe synergy for mechanistically distinct pairs, is expected and informative.

## 4. Results

The pipeline adopted in this study and the main steps are illustrated in Figure 1. The overall process entails the analysis of stage-specific MM-related transcriptomics datasets to identify significant genes, with the subsequent identification and shortlisting of candidate repurposed drugs and the pathways they target.

### 4.1. Differential Expression Analysis

The first part of this study included the collection and analysis of publicly available transcriptomics datasets of MGUS, sMM, and MM patients and controls. Following the pre-processing of these datasets, we performed differential analysis to identify differentially expressed genes (DEGs) between patients in each stage vs. controls. We used a cutoff of adjusted *p* values < 0.05 and then sorted the differentially expressed genes based on their log_2_ fold-change (log_2_FC) value. All differential expression analysis comparisons are presented in Tables S1–S6. The top five differentially expressed genes for each comparison are listed in Table 2.

### 4.2. Pathway Analysis of Differentially Expressed Genes

GO enrichment analysis was performed to detect the statistically significant pathways involving the DEGs from the different datasets per stage. In the MGUS stage, several biological processes (BPs) related to the immune response were significantly detected (Table S7). The highest scores were associated with processes like the adaptive immune response, particularly those involving immune receptor recombination- and leukocyte-mediated immunity. This indicates heightened immune activity at this early stage, where processes such as regulation of leukocyte and lymphocyte proliferation, cell chemotaxis, and immune responses to hydrogen peroxide are prominent. Notably, positive regulation of cytokine production, reactive oxygen species (ROS) metabolic process, and cell–cell adhesion suggest an active environment where immune cells are mobilised and interact to control abnormal cell growth.

In the sMM stage, immune system regulation intensifies, as seen in the overrepresentation of GO terms related to mononuclear and lymphocyte proliferation, positive regulation of cell activation, and T cell differentiation (Table S8). Nearly all immune-related pathways show high scores (0.99), indicating robust immune modulation. Key processes such as immune response-activating cell surface receptor signalling and antigen receptor-mediated signalling pathways are highly active, suggesting a pre-cancerous state where immune surveillance attempts to combat disease progression. Additionally, terms involving positive regulation of leukocyte and lymphocyte adhesion imply strong immune cell communication and activation during this transitional phase.

In the MM stage, there is still emphasis in immune-related processes, with some unique changes (Table S9). The B cell receptor signalling pathway and B cell activation become more prominent, suggesting the involvement of B cells in the disease’s progression. Additionally, cytokine production, particularly interleukin-6 (IL-6), and processes related to leukocyte migration and myeloid leukocyte cytokine production highlight the advanced immune dysregulation. These factors are likely contributing to the chronic inflammatory environment.

Across the three stages (MGUS, sMM, and MM), there is a clear progression in immune response activities. In the MGUS stage, the immune system is highly active with processes focused on immune cell proliferation and activation. As the disease progresses to the sMM stage, the immune system continues to play a central role, with heightened regulation of lymphocyte activation and immune signalling pathways. By the MM stage, however, there is a shift towards immune dysfunction, with an emphasis on B cell activation and cytokine production, particularly IL-6, a known contributor to MM progression. This progression reflects how the immune system, initially attempting to control the abnormal cells, becomes increasingly compromised, allowing for tumour growth and proliferation in later stages. Full lists of pathways for each stage are presented in Tables S7–S9.

### 4.3. Identification of the Shortlisted Candidate Repurposed Drugs for MM and Its Stages

**Figure 2:**
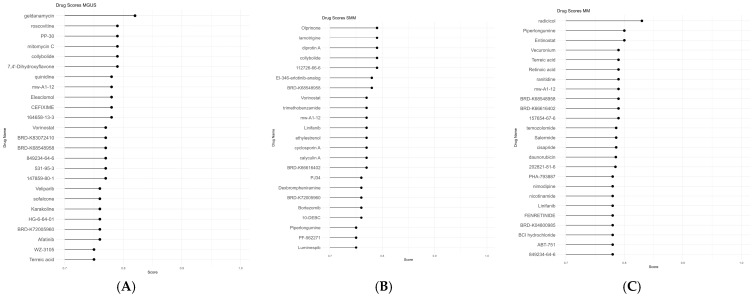
Top-scored candidate repurposed drugs for each disease state (MGUS, sMM, and MM) according to our scoring scheme [23]. (**A**). MGUS, (**B**). sMM, and (**C**). MM. Bortezomib, which is detected in the sMM stage, is an already FDA drug for MM.

To perform in silico drug-repurposing analysis, we selected the top 150 over- and 150 under-expressed genes as they are required for most repurposing tools. Using the DEG sets, we performed a series of in silico drug-repurposing analyses with existing computational tools (see Methods); Connectivity Map (CLUE), L1000CDS^2^, and SigCom LINCS, leading to three lists of candidate repurposed drugs, for each stage and dataset, followed by a scoring process yielding to three lists of proposed repurposed drugs for each stage (MGUS, sMM, and MM). The top repurposed drugs selected are shown in Figure 2. Our in silico drug-repurposing analysis identified several candidate drugs with the potential for repurposing in MM. These drugs were evaluated based on their clinical trial status, preclinical evidence, mechanisms of action, and gene targets (Table S16). While some drugs have been previously investigated in MM, others have only been tested in other cancer types. Key mechanisms of action included cyclin-dependent kinase (CDK) inhibition, histone deacetylase (HDAC) inhibition, and selective estrogen receptor modulation.

For MGUS, the top candidate repurposed drugs proposed regarding the in-house scoring scheme used were geldanamycin, roscovitine, PP-30, mitomycin C, and collybolide. For sMM, the top candidate repurposed drugs proposed regarding the scoring scheme used were olprinone, lamotrigine, diprotin A, collybolide, and 112726-66-6 (BTCP). For MM, the top candidate repurposed drugs proposed regarding the scoring scheme used in this study were radicicol, piperlongumine, entinostat, vecuronium, and terreic acid (Tables S10–S12).

### 4.4. Investigation of Structural Similarity Concerning Ongoing Clinical Trials

**Figure 3:**
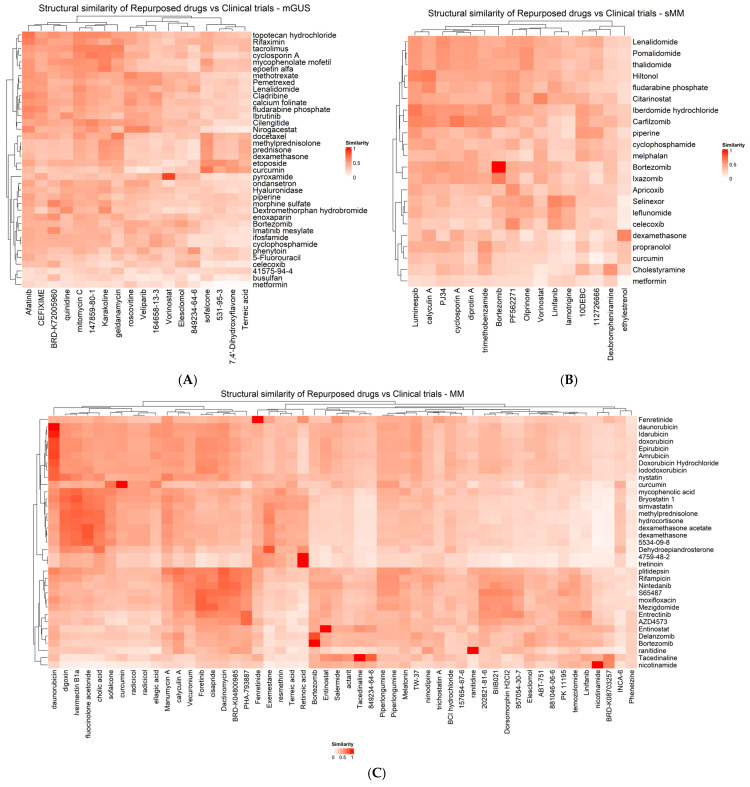
Structural similarity heatmaps of shortlisted repurposed drugs along with the ongoing drugs in clinical trials for (**A**) MGUS, (**B**) sMM, and (**C**) MM. For MM, the maximum similarity between a repurposed drug and a clinical trial drug was kept, since too many drugs were available for this stage. X-axis shows the candidate repurposed drugs. Y-axis shows the drugs in clinical trials for each disease state.

The shortlisted candidate repurposed drugs were screened for structural similarity with the MGUS-, sMM-, and MM-related drugs in clinical trials, and FDA-approved drugs of MM. Pairwise structural similarity was calculated using a Tanimoto score threshold of 80%. As shown in Figure 3, heatmaps for each disease state suggest that the majority of all examined drugs are not very similar, indicating a lack of redundancy and a wide range of structural diversity in both our shortlist and clinical trials. However, some similarities are present, particularly for MM. For instance, ten candidate repurposed drugs are also used in ongoing clinical trials, therefore showing a Tanimoto similarity of 100%. Additionally, the candidate repurposed drug exemestane, was found to have a similarity score of 82% against dehydroepiandrosterone, a clinical trial drug. The repurposed drug fluocinolone acetonide was also found to have a similarity score of 82% against the clinical trial drug dexamethasone. Lastly, the repurposed drug ivermectin b1a showed an 81% similarity with the clinical trial drug bryostatin 1.

**Figure 4:**
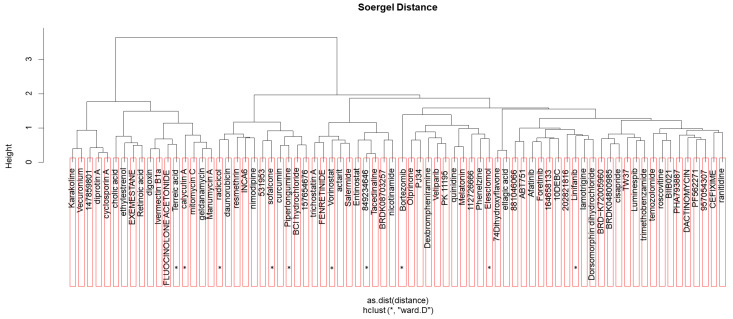
Hierarchical clustering of the shortlisted candidate repurposed drugs for MGUS, sMM, and MM. The different groups in each box are thresholded at Soergel distance value 0.15. Candidate repurposed drugs that are detected in two disease stages are marked with an asterisk (*).

Additionally, the shortlisted candidate repurposed drugs were screened for structural similarity among the three stages: MGUS, sMM, and MM. Pairwise structural similarity was calculated using a Tanimoto score threshold of 87%. As shown in Figure 4, again in this comparison, the hierarchical clustering suggests that all drugs are not very similar, indicating a lack of redundancy and a wide range of structural diversity in our shortlisted candidate repurposed drugs. Ten of these drugs were detected in two disease stages as shown by the asterisk. These drugs include terreic acid, calyculin A, radicicol, sofalcone, piperlongumine, vorinostat, 849234-64-6 (4-acetamido-N-(2-amino-5-thiophen-2-ylphenyl)benzamide), bortezomib, elesclomol, and linifanib.

To further explore the candidate repurposed drugs, we extracted the corresponding gene targets of each drug through mainly the Drug repurposing hub database, and in cases where no target was found, DrugBank and PubChem were also used (Tables S13–S15).

We then performed Gene ontology (GO) analysis (biological processes) per disease stage, using the gene targets of the candidate drugs through the ClusterProfiler R package. Additionally, hierarchical clustering of the enriched terms was performed again using the ClusterProfiler R package. This relies on the pairwise similarities of the enriched terms calculated by the use of Jaccard’s similarity index (JC) (Supplementary Figures S1 and S2).

To further elucidate the functional differences and commonalities between the three disease stages, we generated an enrichment map plot using the ClusterProfiler R package, organising enriched terms into a network with edges connecting overlapping gene sets. In this way, mutually overlapping gene sets tend to cluster together, making it easy to identify functional modules. These gene targets of the proposed candidate repurposed drugs from our analysis, provide valuable insights into the fundamental transcriptional characteristics underlying the functional properties of MGUS, sMM, and MM.

**Figure 5:**
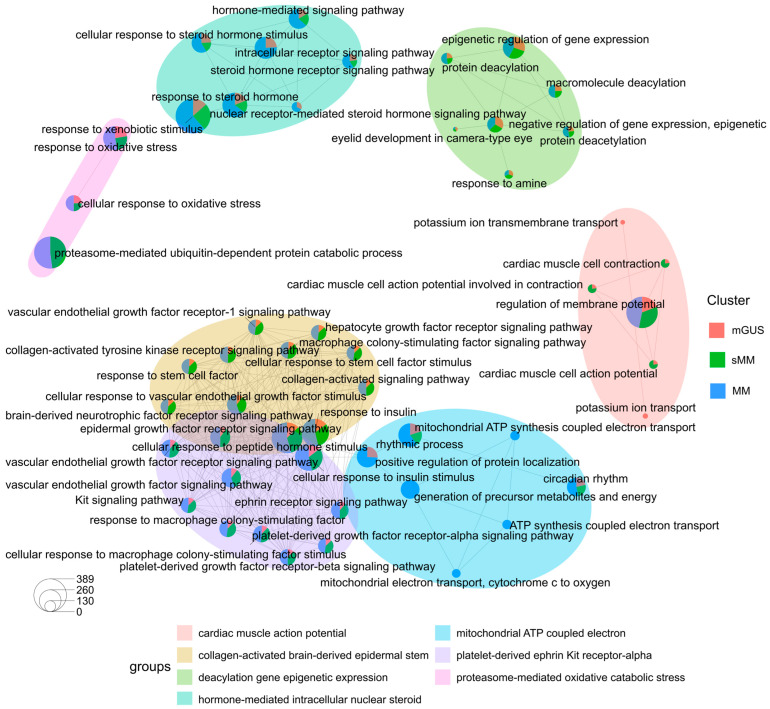
Biological theme comparison of MGUS, sMM, and MM. The enrichment map shows the top enriched terms in MGUS, sMM, and MM, organised into a network with edges connecting overlapping gene sets. Coloured-based clustering was performed using the emapplot function, which reflects functional similarity among enriched terms.

This analysis highlights BPs involved in “cell signalling pathways”, such as vascular endothelial growth factor signalling pathway, brain-derived neurotrophic factor receptor signalling pathway, response to insulin and others (Figure 5, yellow cluster). Moreover, the purple cluster is involved in cellular response to insulin stimulus, cellular response to peptide hormone stimulus, response to macrophage colony-stimulating factor, and others. Notably, the yellow and purple clusters overlapped spatially, indicating that these functionally coherent groups share biologically related terms. Additionally, BPs involved in membrane and action potentials and potassium ion transport were also highlighted (Figure 5, red cluster). GO terms associated with this cluster were highly enriched in all disease stages, and particularly in MGUS and sMM. Another important cluster generated by this analysis highlights BPs involved in epigenetic regulation of gene expression and protein modification (Figure 5, green cluster). Additionally, GO terms associated with this cluster were highly enriched in all disease stages. Moreover, a cluster highlighting Circadian rhythms, protein localisation, mitochondrial function, and others, has also been generated through this analysis. Most BPs of this cluster have been highlighted in MM (blue cluster, Figure 5). The blue-green cluster is focused on hormone-related signalling pathways, with the GO terms associated with this cluster being enriched in all disease stages. Lastly, a small cluster associated with response to oxidative stress has also been highlighted (yellow cluster, Figure 5).

### 4.5. Drug Combination Synergies

The drug combination data were extracted through the DrugComb database (see Methods). In this work, we kept four synergy models: ZIP (Zero Interaction Potency), Loewe Additivity, HSA, and Bliss Independence. Drug combinations are considered synergistic if they exceed specific thresholds: moderate synergy (>+5) and strong synergy (>+10) (Supplementary Figure S3).

### 4.6. Top Drug Combinations Identified

To find the top synergies regarding our disease of interest, MM, a systematic analysis of MM’s FDA-approved drugs was carried out to detect synergies in general and, more specifically, synergies with our proposed candidate repurposed drugs. When looking for drug combinations common across all synergy models, 17 drug combinations were detected (Supplementary Figures S4 and S5), from which, however, none include any of our proposed candidate repurposed drugs. Therefore, we stick to the drug combinations for each synergy model separately, which are then combined in a final list.

**Figure 6:**
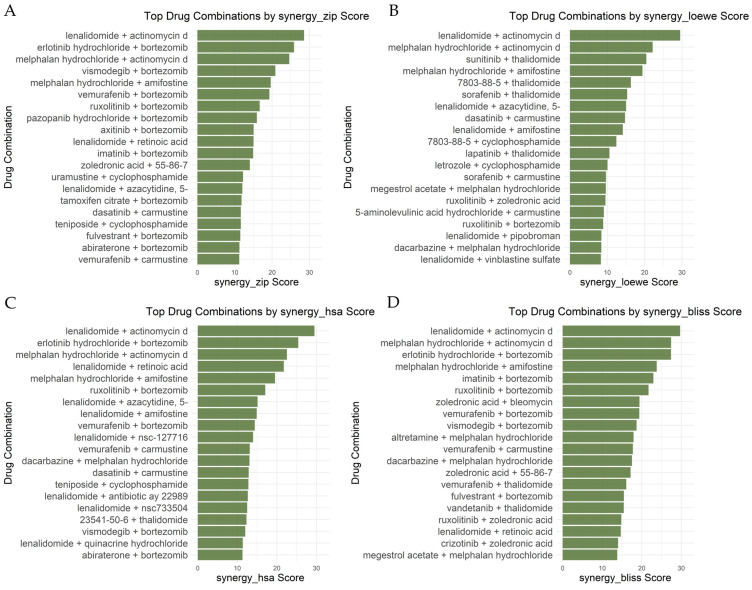
Summary of the top 20 drug combinations per model. The figure presents a comparative analysis of synergy scores across different models, highlighting the most promising combinations for MM treatment. (**A**) Synergy Zip Score model, (**B**) Synergy Loewe Score model, (**C**) Synergy HSA Score model and (**D**) Synergy Bliss Score Model.

A total of 181 drug combinations exhibited synergy across multiple, with some combinations including drugs from our proposed candidate repurposed drug list. The strongest synergies observed per model are shown in Figure 6, in a subset of 20 top-ranking combinations. Notably, an analogue of one of the proposed candidate repurposed drugs, erlotinib, was also identified, further supporting its potential application in combination therapy. Top drug combinations of FDA drugs along with our proposed candidate repurposed drugs include lenalidomide + retinoic acid (from the ZIP and Bliss models), erlotinib hydrochloride + bortezomib (from the ZIP model), and 23541-50-6 (daunorubicin HCL) + thalidomide (from the HAS model). The total significant drug combinations can be found in Table S19.

The results suggest that several repurposed drugs could be explored further for their potential in MM treatment. Additionally, the identification of highly synergistic drug combinations underscores the importance of combination therapy approaches. Future studies should focus on validating these findings in preclinical and clinical settings to assess their therapeutic potential.

## 5. Discussion

The search for effective treatments for MM continues to be a substantial problem, requiring ongoing investigation into new therapeutic approaches. Drug repurposing, the process of using existing drugs for a novel disease, offers a promising approach due to its cost- and time-effective nature. Drug repurposing is essential in the advancement of innovative anti-cancer therapies. This study explores the capabilities of transcriptomic signature-based drug repurposing using all the publicly available bulk transcriptomics datasets on MGUS, sMM, and MM. We filter and prioritise candidate repurposed drugs to be shortlisted for further analysis in the future. Our study included a scoring scheme from a previous work of our group [23], resulting in 25 candidate repurposed drugs for MMGUS, 23 for sMM, and 66 for MM. From these, 18 candidate repurposed drugs for MGUS, 16 for sMM, and 52 for MM had available structure information. These highlighted drugs have generally been shown to be structurally distinct from each other, meaning that most of the proposed drugs do not belong to the same structural subgroup. Additionally, Gene ontology terms (biological processes) were detected using the DEGs of each dataset and disease state. The same scoring scheme used for the candidate repurposed drugs was also used here to select a single list of pathways for each disease state. Lastly, we detected the gene targets of the proposed repurposed drugs along with the associated Gene ontology terms (biological processes).

According to MeSH, when analysing the top 15 proposed candidate repurposed drugs for each disease stage—MGUS, sMM, and MM—three drugs were characterised as antineoplastic for MGUS, including geldanamycin, mitomycin, and roscovitine. Additionally, for sMM, two drugs were detected as antineoplastic: linifanib and vorinostat. Lastly, for MM, five out of the fifteen top proposed candidate repurposed drugs were antineoplastic agents, including radicicol, entinostat, salermide, temozolomide, and daunorubicin. Other drug categories among the top proposed candidate repurposed drugs include immunosuppressants (e.g., cyclosporin A detected for sMM).

To synthesise the pathway-level signals, we summarise key therapeutic families implicated by our stage-specific targets and drug candidates (Table 3). This table highlights where candidates align with known mechanisms versus under-explored processes enriched in earlier stages.

Many of the pathways detected using the gene targets of the proposed candidate repurposed drugs are involved in cancer. For instance, pathways detected in the “yellow and purple cluster” (Figure 5), such as Kit signalling pathway and ephrin receptor signalling pathway, are involved in cellular processes like proliferation, differentiation, controlling cell growth, survival, and metabolism. Specifically, the former is a cellular signalling cascade initiated by the binding of stem cell factor (SCF) to the KIT receptor, which is a tyrosine kinase, that when activated leads to the activation of multiple signalling pathways. These include MAPK, PI3K, and JAK/STAT, which regulate diverse cellular processes like cell growth, survival, migration, and differentiation [54]. Additionally, the latter, the ephrin receptor signalling pathway, is a key cell-to-cell communication system involved in various cellular processes, such as cell migration [55]. In MM, various cytokines and cell adhesion molecules are the key players in helping MM cells and the bone marrow microenvironment to interact. This interaction promotes the activation of signalling pathways such as PI3K/AKT/mTOR, RAS/MAPK, JAK/STAT, Wnt/β-catenin, and NF-κB. In the case of MM, these pathways are aberrantly activated, and, therefore, uncontrolled proliferation, survival, migration, and drug resistance of myeloma cells persist. Hence, these pathways are great therapeutic targets [56]. Additionally, the vascular endothelial growth factor pathway (VEGF), which was also detected in the aforementioned cluster, was shown to be essential in MM cell migration to the bone marrow and peripheral blood, and also in cancer metastasis [57,58].

Moreover, regarding the “red” cluster, ion channels are involved in several biological processes, including proliferation, cell volume and shape, differentiation, migration, and apoptosis, and have been recently associated with malignant transformation, tumour progression, and drug resistance. Specifically, for MM, it has been shown that MM cells’ survival, proliferation, and drug resistance can be a result of ion channel deregulation. For instance, in MM settings, changes in K^+^ currents modify membrane potential and affect intracellular pathways that regulate cell proliferation. Additionally, treatment with arsenic trioxide, which reduces voltage-gated potassium currents and affects cell activity based on dosage, slows the growth of MM cells by stopping them in the G0/G1 phase. Wu and colleagues also showed that using berberine, a natural compound, on MM cells blocks potassium currents and limits their growth [59]. Moreover, epigenetic regulation is another key process, both in MM and in several cancer types. In MM, key epigenetic processes like DNA methylation and histone acetylation are known to contribute to the disease’s development. Recently, research has also introduced the term “epi-microRNAs,” which refers to microRNAs that can influence these epigenetic processes by targeting specific regulators such as DNA methyltransferases and histone deacetylases [60].

Steroid hormone signalling, another process detected through our candidate repurposed drugs, can directly stimulate the MEK/ERK/RSK pathway to regulate cellular proliferation and survival in transformed cells [61]. Furthermore, response to oxidative stress and ROS were also detected through our analysis. In most stages of MM, there is an increase in free radicals and a disruption of the body’s antioxidant system, which causes the cancer cells to grow rapidly. However, in advanced stages accompanied with other comorbidities, reducing oxidative stress can lead to further tumour growth. New treatments for MM, like proteasome inhibitors, immunomodulatory drugs, epigenetic drugs, and monoclonal antibodies, have helped improve survival and quality of life. These treatments increase oxidative stress, leading to cancer cell death by triggering a process known as the Unfolded Protein Response [62].

Interestingly, our analysis showed that the ROS metabolic process was detected only in MGUS but not in sMM or MM. This suggests that MGUS cells may have an active system to manage ROS levels, which could help them maintain stability and prevent damage. In contrast, MM cells are known to experience high oxidative stress without activating the relevant ROS degradation processes. The loss of ROS regulation in MM may contribute to disease progression by allowing harmful oxidative damage to accumulate. This finding highlights a potential shift in how cells handle oxidative stress as the disease advances, which could be important for understanding why some MGUS cases progress to MM while others remain stable. Further studies could help determine whether targeting redox balance might be useful for preventing or slowing disease progression [62,63].

The detection of the ubiquitin-dependent protein catabolic process exclusively in sMM and MM aligns with the current understanding of MM pathology. The ubiquitin–proteasome system (UPS) is integral to cellular protein degradation, regulating processes such as cell cycle progression, apoptosis, and differentiation. In MM, dysregulation of the UPS contributes to uncontrolled cell proliferation and survival. Proteasome inhibitors like bortezomib have been developed to target this pathway, inducing apoptosis in MM cells by disrupting protein degradation mechanisms. The exclusive activation of the ubiquitin-dependent protein catabolic process in sMM and MM, but not in MGUS, suggests a pivotal role of UPS dysregulation in the progression and maintenance of malignant phenotypes in MM [64,65].

There are different ways to measure drug synergy. Each method, however, has its strengths and limitations. The four models used in this study—ZIP, Loewe, HSA, and Bliss—evaluate synergy in different ways. First, Loewe Additivity is the strictest model since it assumes that two drugs with similar effects should act additively. As a result, it often gives lower synergy scores and may underestimate synergy. However, it is useful for avoiding false positives, making it a good choice for verifying strong synergies. Second, ZIP is a more balanced model. It considers dose–response relationships, meaning it detects synergy that occurs across different concentration levels. It is less strict than Loewe but still prevents extreme synergy overestimations. Additionally, HSA is more lenient since it only compares the combination to the most effective single drug. If the combination outperforms one of the two drugs, it is considered synergistic. This method tends to find more synergy but may also produce some false positives. Lastly, Bliss Independence assumes drugs work independently and measures synergy based on probabilities. It is the most sensitive method, often detecting the highest synergy values, which can sometimes lead to overestimated synergy scores.

Based on the results of this study, Loewe produced the most conservative scores, while Bliss and HSA detected higher synergy values. ZIP provided a balanced approach. Because Loewe detected many negative scores and outliers, it may not be the best choice for selecting promising drug combinations. Instead, combinations that show strong synergy across ZIP, HSA, and Bliss models may be more reliable. This approach helps reduce false positives while still capturing meaningful interactions.

Future work should include integration of stage-aware transcriptomics with proteomics, metabolomics, and/or single-cell/spatial data. Additionally, the most promising stage-specific candidates should be tested experimentally: (i) assess single-agent activity across a small MM cell-line panel and (ii) assess drug combinations using the FDA-approved drugs as backbones along with our shortlisted candidate repurposed drugs.

## 6. Conclusions

We have identified a shortlist of candidate repurposed drugs inferred from analysis of publicly available bulk RNA data, in a stage-specific manner. We derive the most enriched pathways detected through our differentially expressed genes, and through the gene targets of our top candidate repurposed drugs we detected the most enriched pathways targeted by them. Lastly, we detect which are the structurally similar candidate repurposed drugs to the current clinical trials for MM and its preceding stages. Lastly, we propose drug combinations of the FDA-approved drugs of MM along with our proposed repurposed drugs. While MM is a complex disease, we anticipate that our proposed drug candidates, along with the drug combinations, offer valuable molecular insights and highlight promising directions for future therapeutic development. Figure S5. Differences in model behaviour. A. Boxplots of synergy scores across the four models, illustrating their respective ranges, median values, and outliers. B. The heatmap displays synergy scores for individual drug combinations across all four models. It shows the highest (red) to lowest (blue) synergy scores observed across the n different models.

## Figures and Tables

**Table 2:** The top 5 differentially expressed genes for each comparison.

	MGUS	sMM	MM
GSE36474			MAB21L1 XG EMX2 HOXB-AS3 FAM3A
GSE5900	KIT IGLC1 PRR15 C7orf55 LOC100293211	KIT CYAT1 LOC100293211 IGHV3-73 C7orf55	
GSE6477	CLC PRG2 LOC100293211 RNASE2 PRG3	IGHD IGLJ3 LOC100293211 CKAP2 IGHA1	IGHD IGLJ3 LOC100293211 CKAP2 IGHA1
GSE13591	IGLV1-44 IGLC1 LOC100293211 IGK CKAP2		IGHD LOC100293211 AbParts IGLV1-44 IGHM
GSE47552	IGKV2D-40 SNORD115-1 SNORD115-6 GPR15 SNORD115-44	IGKV2D-40 IGKV2D-26 IGKV1D-27 IGHV1OR15-1 IGKV1OR2-3	IGKV2D-40 IGKV2D-26 IGKV1OR2-3 IGKV6-21 IGKV1D-27
GSE80608	SFRP2 H19 SLC14A1 F2R SCIN		FLG H19 SLC14A1 F2R SCIN

**Table 3:** Mechanistic families for shortlisted repurposing candidates across the MGUS, sMM, and MM spectrum.

Pathway Family	Representative Targets/Mechanisms	Example Candidates from Our Shortlist	Key References
HSP90/proteostasis	HSP90AA1/HSP90AB1 chaperoning of oncogenic clients; stabilisation of FOXM1; stress-survival under hypoxia/chemo	Geldanamycin, Radicicol, Luminespib, Radicicol, and derivative KF55823	[35,36,37,38,39]
DNA damage—response effectors	DNA damage response activation; topoisomerase II inhibition; CDK2/7/9 blockade; apoptosis with MCL1 down-regulation; ↓ IL-6 transcription/expression; metabolised to an alkylating agent	Daunorubicin, Seliciclib (Roscovitine), CGP-60474, and Mitomycin C	[40,41,42,43,44,45]
Oxidative stress	↑ ROS leading to mitochondria-dependent apoptosis; direct inhibition of STAT3 (Cys712)	Piperlongumine	[46,47,48]
Epigenetic modulation—HDAC inhibition	HDAC1/2/6 inhibition; epigenetic reprogramming; synergy with proteasome inhibition	Entinostat (MS-275/SNDX-275)	[49], NCT00015925
Neuromuscular nicotinic AChR antagonism	Muscle-type nAChR (CHRNs) blockade; preclinical anti-metastatic effects with cisatracurium	Vecuronium bromide	[50,51]
BTK/B cell receptor signalling	BTK catalytic inhibition; impacts mast-cell activation and B cell development	Terreic acid	[52,53]

## Supplementary Materials

The following supporting information can be downloaded at: https://www.mdpi.com/article/10.3390/cancers17183045/s1, Figure S1. GO enrichment analysis of biological processes (BPs) of MMGUS sMM and MM. The transition in colour from red to blue represents the adjusted *p*-value, while the size of the dot is proportional to the number of genes enriched in that particular process or pathway. Figure S2. Heatmap of hierarchical clustering of enriched terms for the three clusters (MGUS, sMM and MM). Circle sizes indicate gene number per term. The enriched GO terms were further clustered in different colours. The subtrees are labelled with high-frequency words. The clustering was generated using the ClusterProfiler R package. Figure S3. Distribution of synergy scores across different drug combinations. These histograms categorise combinations based on their synergy strength to illustrate the frequency of highly synergistic interactions. Figure S4. Commonalities among the synergy models used in the analysis. The figure highlights the overlap between models and the number of drug combinations identified as synergistic by multiple models. Figure S5. Differences in model behaviour. A. Boxplots of synergy scores across the four models, illustrating their respective ranges, median values, and outliers. B. The heatmap displays synergy scores for individual drug combinations across all four models. It shows the highest (red) to lowest (blue) synergy scores observed across the n different models. Table S1: differential expression analysis of the GSE36474 dataset. Differentially expressed genes were selected based on a log fold change (log2FC) threshold of ≤ −1 and ≥ 1, and an adjusted *p*-value (adj. p) ≤ 0.05. Table S2: differential expression analysis of the GSE5900 dataset. Differentially expressed genes were selected based on a log fold change (log2FC) threshold of ≤ −1 and ≥ 1, and an adjusted *p*-value (adj. p) ≤ 0.05. Table S3: differential expression analysis of the GSE6477 dataset. Differentially expressed genes were selected based on a log fold change (log2FC) threshold of ≤ −1 and ≥ 1, and an adjusted *p*-value (adj. p) ≤ 0.05. Table S4: differential expression analysis of the GSE13591 dataset. Differentially expressed genes were selected based on a log fold change (log2FC) threshold of ≤ −1 and ≥ 1, and an adjusted *p*-value (adj. p) ≤ 0.05. Table S5: differential expression analysis of the GSE47552 dataset. Differentially expressed genes were selected based on a log fold change (log2FC) threshold of ≤ −1 and ≥ 1, and an adjusted *p*-value (adj. p) ≤ 0.05. Table S6: differential expression analysis of the GSE80608 dataset. Differentially expressed genes were selected based on a log fold change (log2FC) threshold of ≤ −1 and ≥ 1, and an adjusted *p*-value (adj. p) ≤ 0.05. Table S7: Merged pathway rankings according to our scoring scheme (see methods) for MGUS. Table S8: Merged pathway rankings according to our scoring scheme (see methods) for sMM. Table S9: Merged pathway rankings according to our scoring scheme (see methods) for MM. Table S10: All candidate repurposed drugs proposed for mgus according to our scoring scheme for MGUS. Table S11: All candidate repurposed drugs proposed for mgus according to our scoring scheme for Smm. Table S12: All candidate repurposed drugs proposed for mgus according to our scoring scheme for MM. Table S13: Top candidate repurposed drugs proposed for MGUS along with the drug targets. All drug targets were extracted from the Drug repurposing hub. Table S14: Top candidate repurposed drugs proposed for sMM along with the drug targets. All drug targets were extracted from the Drug repurposing hub. Table S15: Top candidate repurposed drugs proposed for MM along with the drug targets. All drug targets were extracted from the Drug repurposing hub. Table S16: Top proposed candidate repurposed drugs containing information on clinical trials and pre-clinical studies, and also mechanisms of action of the drugs. Table S17: Detailed synergy scores for each drug combination, before any threshold cut-offs. Table S18: All commonalities presented in the Venn diagram across the different models. Table S19: Results from DrugComb database on synergies of the FDA drugs. Highlighted in orange colour are the combinations that contain drugs that were also detected from our proposed repurposed list. In green, a drug is highlighted for which an analogue was identified among our proposed candidate repurposed drugs.
