# Supplementary material for: Computational Drug Repurposing Across the Multiple Myeloma Spectrum: From MGUS to MM
Source: Cancers (Basel). 2025 Sep 18;17(18):3045. doi: 10.3390/cancers17183045 (PMC12468375; doi:10.3390/cancers17183045)
Supplement: Supplementary file 1 [file cancers-17-03045-s001.zip › cancers-3824174-supplementary.pdf]

Supplementary text and figures

Enrichment analysis using gene drug targets

We performed Gene ontology (GO) analysis (Biological processes) per disease stage, using the gene targets of the candidate drugs through the ClusterProfiler R package. In Figure S1, Gene Ontology (GO) analysis indicated the involvement of the three stages in various common biological processes such as *cellular response to oxidative stress, positive regulation of kinase activity, response to steroid hormone, response to oxidative stress, hormone-mediated signalling pathway, response to xenobiotic stimulus, negative regulation of gene expression, epigenetic, protein diacylation and deacetylation, rhythmic process, positive regulation of transferase activity, regulation of membrane potential, muscle system process* and *macromolecule deacylation*. Additionally, several biological processes were found in one or two stages only, such as *potassium ion transport,* which was found only in MGUS. Moreover, the *ubiquitin-dependent protein catabolic process* was the only BP detected in stages sMM and MM. *Mitochondrial electron transport, cytochrome c to oxygen and one-carbon metabolic process* were detected only in the stage of MM. Lastly, *intracellular receptor signalling pathway* was detected only in MGUS and MM.

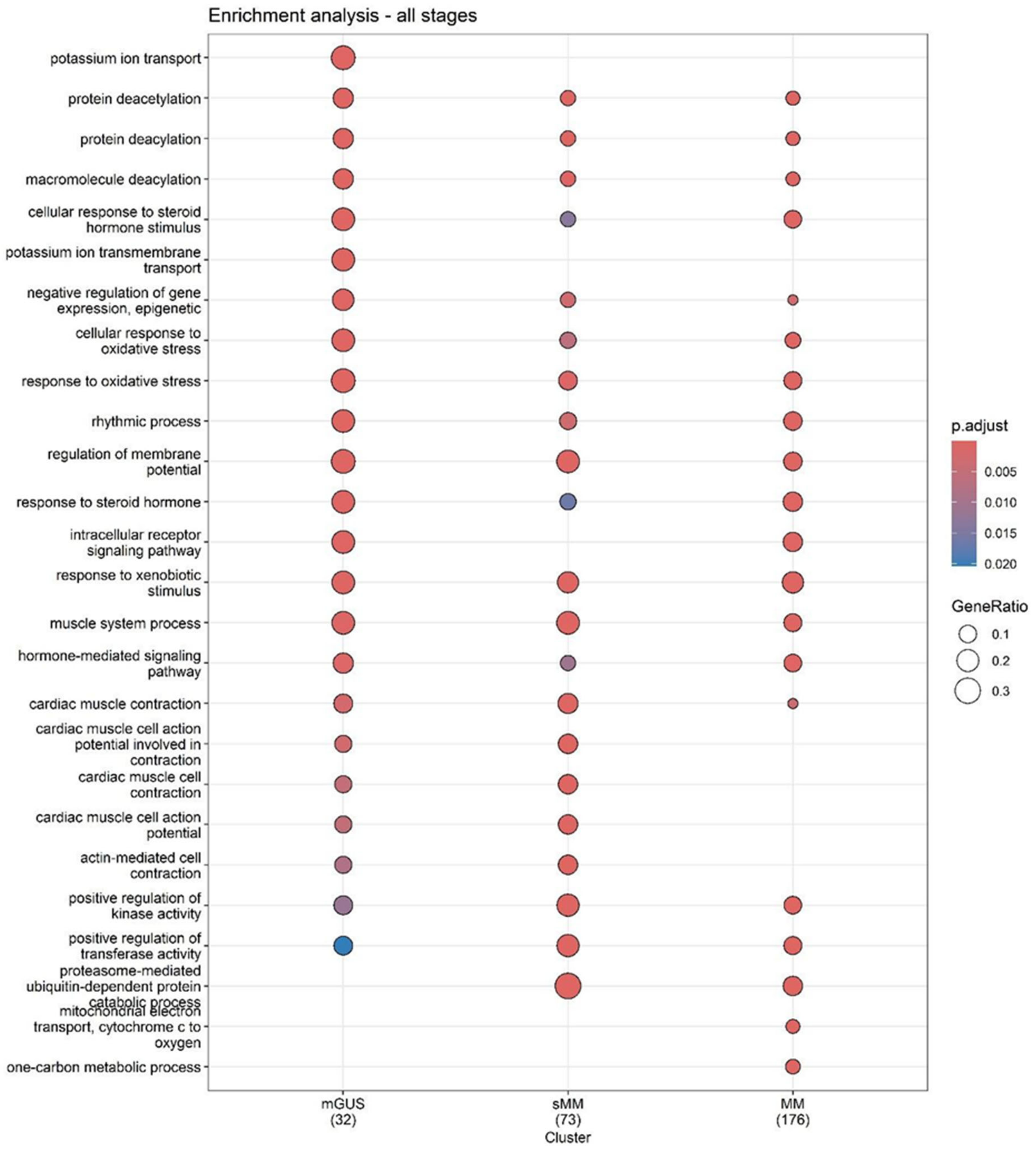

Figure S1. GO enrichment analysis of biological processes (BPs) of MGUS sMM and MM. The transition in colour from red to blue represents the adjusted p. value, while the size of the dot is proportional to the number of genes enriched in that particular process or pathway.

Additionally, hierarchical clustering of the enriched terms was performed using the ClusterProfiler R package. This relies on the pairwise similarities of the enriched terms calculated by the use of Jaccard's similarity index (JC). Again, hierarchical clustering was performed for the three stages of the disease, MGUS, sMM and MM. The enriched terms were hierarchically

clustered in functional families such as BPs related to "protein and macromolecule diacylation/deacetylation", "positive regulation kinase activity", "cellular/intracellular steroid hormone" related to all three disease stages, as shown in Figure S2.

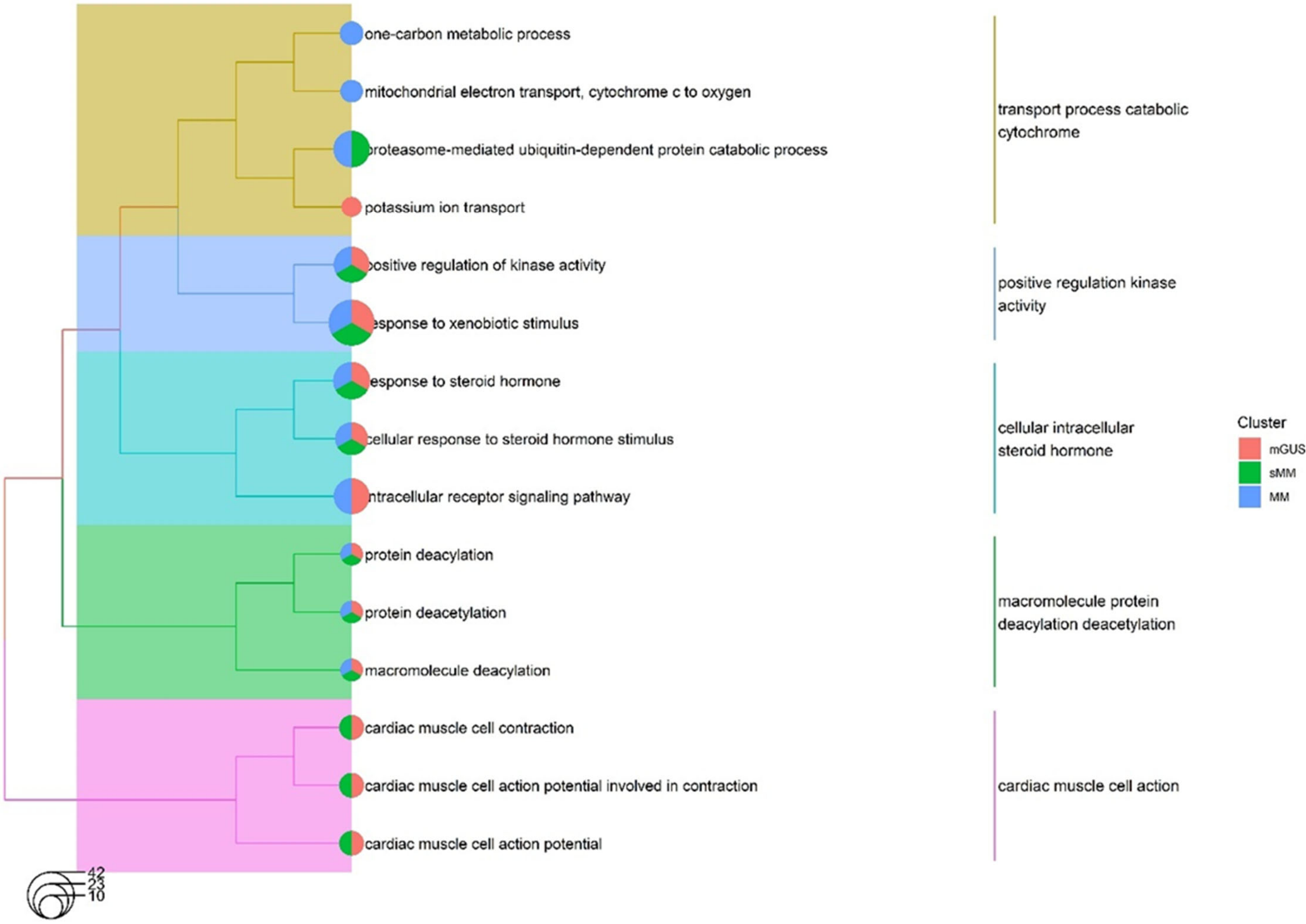

Figure S2. Heatmap of hierarchical clustering of enriched terms for the three clusters (MGUS, sMM and MM). Circle sizes indicate gene number per term. The enriched GO terms were further clustered in different colours. The subtrees are labelled with high-frequency words. The clustering was generated using the ClusterProfiler R package.

Drug Combinations

To choose a threshold for the synergy models regarding our data, we visualised the distributions of each synergy model (ZIP, Loewe, HSA, and Bliss) to decide on the best value (**Figure S3**). Based on the plots, we chose the threshold of >+5 (moderate synergy) from the distribution plots. All synergy scores are shown in **Table S17.**

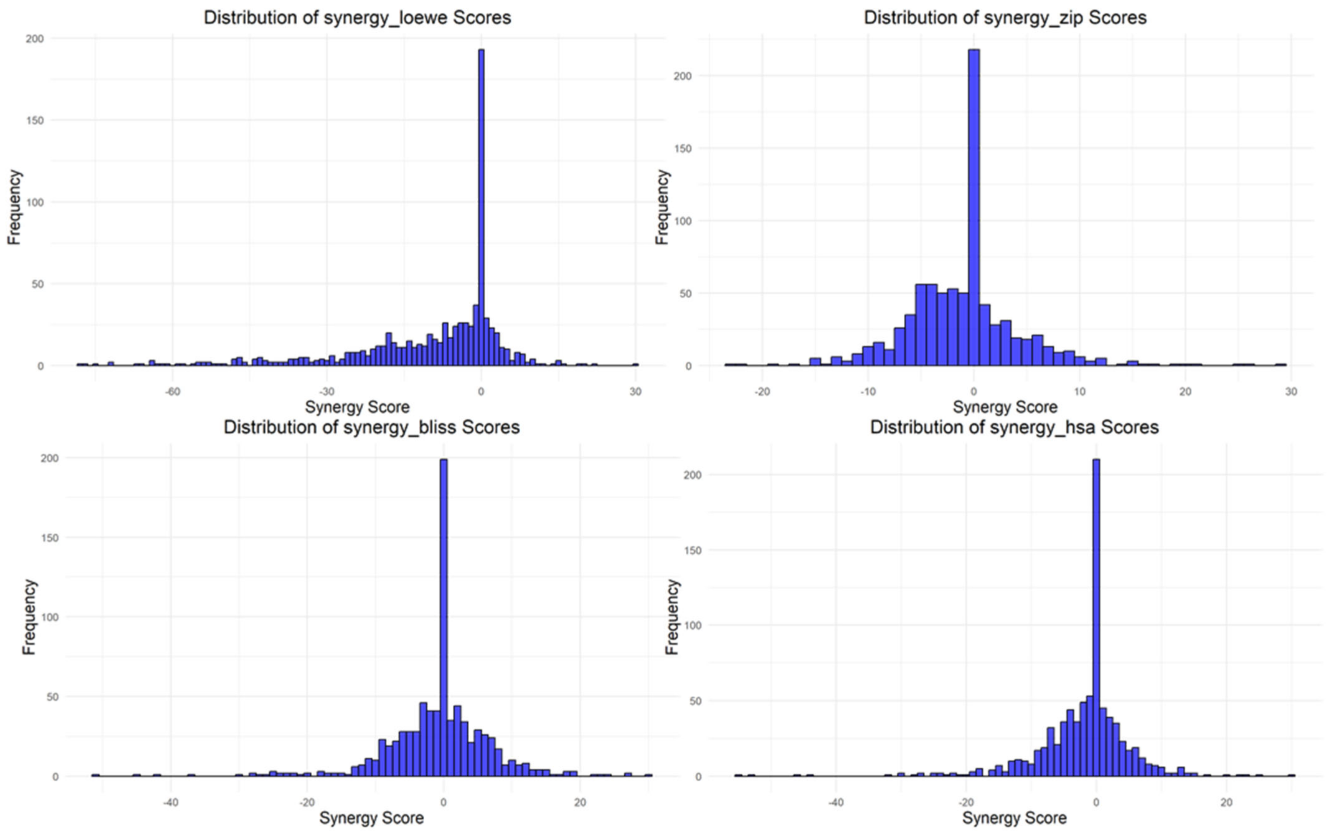

Figure S3. Distribution of synergy scores across different drug combinations. These histograms categorise combinations based on their synergy strength to illustrate the frequency of highly synergistic interactions.

Commonalities Among Synergy Models

The strongest synergies from the analysis were examined across the four synergy models to determine their commonalities. 17 common drug combinations were detected among all four synergy models, reinforcing their potential therapeutic value (**Figure S4**). The majority of commonalities were found among ZIP, HSA and Bliss (33 commonalities). The overlap among the models indicates the robustness of the identified drug combinations. Among the commonalities of the three aforementioned models we find thalidomide with daunorubicin HCl, cyclophosphamide with daunorubicin HCl, and melphalan HCL with mitomycin C. Additionally, the melphalan HCL with daunorubicin HCL combination was detected through the Bliss model. Lastly, the drug combination bortezomib with temozolomide was detected through the ZIP model. All overlaps are presented in **Table S18.**

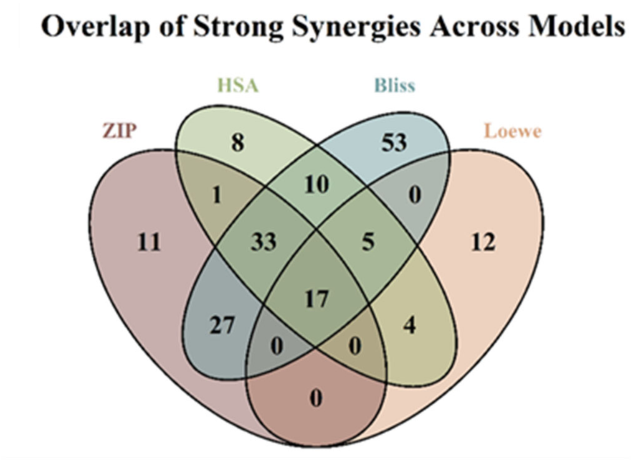

Figure S4. Commonalities among the synergy models used in the analysis. The figure highlights the overlap between models and the number of drug combinations identified as synergistic by multiple models.

Comparison of Synergy Scores Across Models

The distribution of synergy scores was further examined using boxplots for each model, highlighting the range and variability in scores. Loewe exhibited the broadest distribution, with a significant number of negative scores and extreme outliers, suggesting that it is more conservative in defining synergy. However, this model could potentially help identify false positives detected by other models. In contrast, ZIP and HSA models had a more compact range, with synergy scores clustering around zero. Bliss and HSA models tended to capture higher synergy values, suggesting that they may be more sensitive to detecting synergistic interactions (**Figure S5A**). To further explore how synergy scores vary across different models, we generated a heatmap comparing the synergy values assigned to each drug combination (**Figure S5B**). This heatmap confirms the trends shown in **Figure S5A**, where Bliss and HSA tend to detect higher synergy values, while Loewe shows more negative scores. Together, **Figure S5** illustrates the differences in model behaviour and helps prioritise drug combinations that consistently demonstrate high synergy across multiple scoring approaches.

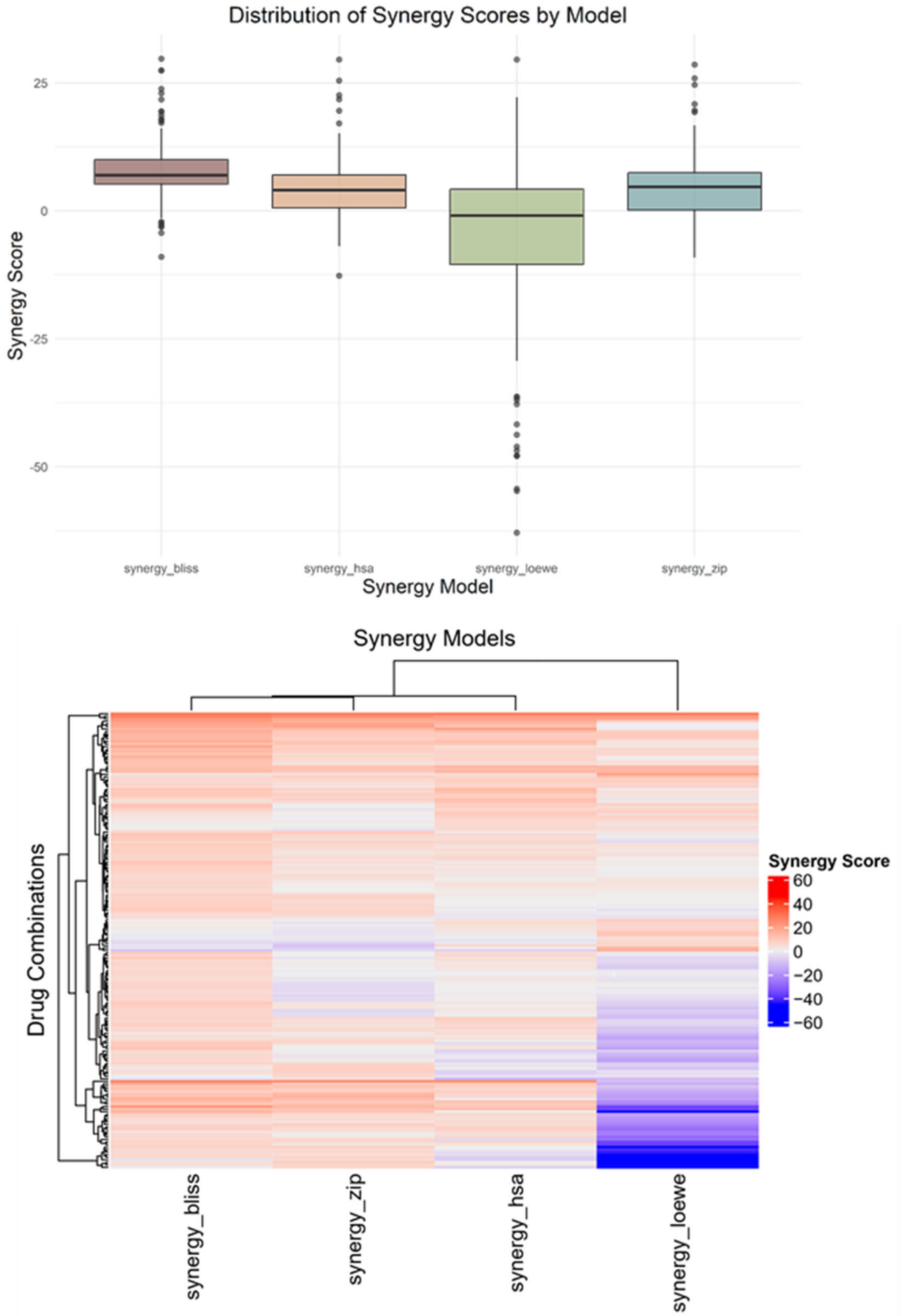

Figure S5. Differences in model behaviour. A. Boxplots of synergy scores across the four models, illustrating their respective ranges, median values, and outliers. **B.** The heatmap displays synergy scores for individual drug combinations across all four

models. It shows the highest (red) to lowest (blue) synergy scores observed across the n different models.
